# Information adoption behavior in online healthcare communities from the perspective of personality traits

**DOI:** 10.3389/fpsyg.2022.973522

**Published:** 2022-10-18

**Authors:** Yiping Zhu, Hong Jiang, Zan Zhou

**Affiliations:** School of Public Policy and Administration, Nanchang University, Nanchang, China

**Keywords:** adoption behavior, personality traits, online community, influential factors, healthcare

## Abstract

Improving standards of living have resulted in an increased focus on health and image management. In a context where the quality of healthcare information is unguaranteed, the adoption behavior intention of online health information varies greatly. Hence, it is essential to take effective measures to guide community users to obtain high-quality information on demand. From the perspective of personality traits, the present study analyzed the influencing factors and mechanisms of adoption behavior intention of healthcare information in online healthcare communities as well as the moderating effects of social support. A quantitative analysis of 380 respondents revealed positive associations between five personality dimensions and the adoption behavior intention of healthcare information–extraversion, agreeableness, conscientiousness, neuroticism, and openness. The study also determined that health concerns and health-related self-efficacy played a mediating role across various degrees between the conscientiousness and adoption behavior intention of healthcare information. As an important contextual factor influencing health outcomes, social support is common in online healthcare communities. The study examined the effect of the interaction between inner traits and social support on adoption behavior intention. Perceived self-esteem support strengthened the indirect effect of conscientiousness on adoption behavior intention mediated by health concerns and health-related self-efficacy. Additionally, the impact of high neuroticism interacted with low levels of perceived self-esteem support on adoption behavior intention was significant. Likewise, emotional supportive information did not help in facilitating the adoption behavior intention in terms of all five personality traits and negatively influence the adoption behavior intention for individuals high in neuroticism and agreeableness. The possible explanation for the results was discussed with the intention of understanding the psychological mechanisms which guide adoption behavior intention.

## Introduction

With the modernization of society, the scale of chronic diseases is increasing in population having a lower age. Nearly 300 million people are suffering from chronic diseases in China, with more than 200 million people experiencing obesity or sleep disorders. Meanwhile, the awareness for healthcare is gradually increasing among citizens. The number of individuals reading about “insomnia” is on the rise, and interest in health-promoting contents, such as organic foods, healthcare products, and physical exercise is escalating. Notably, losing weight has become a trend, and the number of fitness centers is growing in response to it. Disease prevention and control, diet and nutrition, lifestyle habits, fitness, and mental health are the topics most people often discuss. Online healthcare communities, i.e., platforms where a group of people suffering from similar health issues can communicate and interact with each other, are flourishing. Many medical practitioners use online healthcare communities to popularize medical scientific information (Dawson, 2010), and people without medical education are turning to healthcare communities to promote health. Online communities have become an important interactive platform for health information dissemination. At the same time, some scholars have raised concerns about the quality of online health information (Lim and Kim, 2012; Ragusa and Crampton, 2019). In this context, it is necessary to study the factors that influence the adoption behavior intention of healthcare information in online communities to obtain healthcare information more efficiently.

Adoption behavior intention in this study refers to agreeing or believing what is stated in the information, and the likelihood of sharing or acting on what it suggests. The process of information adoption is an interaction between the information receiver, the information itself, and the environment. Numerous researchers have examined the information characteristics of the factors influencing information adoption behavior intention. Among these factors, the Information Adoption Model, Unified Theory of Acceptance and Use of Technology, and Technology Acceptance Model together with its improved version are the most widely used theoretical models. A series of studies have validated the positive impact of the perceived usefulness of information on user acceptance in the TAM model (Venkatesh and Morris, 2000; Tseng and Kuo, 2014; Ahadzadeh and Sharif, 2017; Lee et al., 2020). Information quality and source credibility are key factors regulating user adoption of online information (Shen et al., 2016; Lin et al., 2017; Lai and Liu, 2020). The dimensions of information quality differ from one field to another; nonetheless, in the field of e-commerce, timeliness, trustworthiness, relevance, comprehensiveness of information, background homophily, and attitude homophily are considered integral components of information quality (Cheung et al., 2008; Cheung, 2014; Shen et al., 2016). For online tourism information, the usefulness, playfulness of tourism information, and social influence of the website are the key factors that determine the level of usage of tourism information (Chang and Caneday, 2011; No and Kim, 2014). Furthermore, online medical information, perceived severity, perceived susceptibility, perceived credibility and perceived personal relevance are expected to affect the acceptance of users (Smerecnik et al., 2010; Kim et al., 2012; Puspitasari and Firdauzy, 2019).

In addition to information characteristics, some studies have also assessed the impact of individual differences, such as dispositions and traits, on information adoption behavior intention. It has been determined that information literacy will influence the users’ trust in the website (Lee et al., 2020). On the other hand, trust positively affects adoption behavior intention through information relevance and information reliability (Lim and Kim, 2012). IQ and subjective health knowledge also exert strong indirect effects on individuals’ decisions and actions (Gatian, 1994; Palmer, 2002; Kim et al., 2012), especially in the field of health. Self-efficacy is strongly associated with perceived ease of use (Venkatesh and Morris, 2000), and it can affect users’ behavioral intention to adopt information (Kim et al., 2011, 2012; Humaidi et al., 2018). In terms of personality characteristics, people with high avoidance orientation are more likely to be persuaded by the high-threat message (van’t Riet et al., 2012). Conscientiousness and extraversion traits have a significant positive impact on the actual use of technology closely linked with adoption behavior intention, while neuroticism harms the actual use (Barnett et al., 2015; Shropshire et al., 2015).

Moreover, associations between personality traits and other information behaviors are closely correlated with the behavioral intention for information adoption. Therefore, findings on the relationship between personality traits and other information behaviors are expected to help examine how personality traits influence the adoption behavior intention and interpret the findings. Specifically, personality traits influence information-seeking behavior (Stokes and Urquhart, 2011), including the preferences for online search channels (Jani et al., 2014; Zhang et al., 2017) and the type of information source (Heinstrom, 2003a). People who are high in neuroticism tend to believe and use the paper resources in the libraries, and people who are high in conscientiousness prefer precise and comprehensive information sources (Frechette et al., 2017). There are differences between various personality traits concerning the ability to perform specific information-seeking tasks (Al-Samarraie et al., 2017). Consequentially, personality traits are significantly correlated with information-sharing behavior (Matzler et al., 2008); they can predict users’ willingness to share their health problems on social platforms (Cho, 2017). There is also a strong association between personality traits and information adoption (Kim, 2017). Personality and emotion can affect the likelihood of accidental information acquisition and receptivity to information (Heinström, 2006). Particularly, people with different levels of externality would choose different methods of knowledge acquisition (Akhavan et al., 2016).

Nevertheless, the influence of personality traits on information adoption has not been thoroughly discussed and appraised. The present study adopts the most widely used personality trait model, the five-factor model, to explore the influence of each personality trait on adoption behavior intention in healthcare communities. This paper can provide new research ideas for theoretically investigating the mechanism of information adoption behavior intention.

## Theoretical hypotheses and research models

### The impact of personality traits on the adoption behavior intention of healthcare information

Traits are the fundamental elements of personality. Goldberg’s five-factor theory of personality is developed into extraversion, agreeableness, conscientiousness, neuroticism, and openness to experience of the Big Five personality theories. Extraversion reflects a tendency toward interpersonal relationships, representing enthusiasm, vitality, self-confidence, and gregariousness. Individuals with high levels of such trait are energetic and are likely to communicate and express themselves. They have several friends (Amichai-Hamburger and Vinitzky, 2010); more specifically, they can obtain information from the ever-increasing social resources from which they can reasonably be assumed to elicit advantages concerning information collection, and thus depict a trend of adoption. We propose the following hypothesis:

H1: Extraversion is significantly and positively associated with the adoption behavior intention of healthcare information.

Agreeableness represents altruistic traits such as trust, obedience, and easygoingness. Individuals with high levels of agreeableness tend to be overly considerate of others and lack independent thought. Therefore, agreeable people are easily misled and confused by various types of information. For this reason, individuals who scored higher on the trait of agreeableness tend to adopt a trusting attitude toward the healthcare information posted by other members of communities. We propose the following hypothesis:

H2: Agreeableness is significantly and positively associated with the adoption behavior intention of healthcare information.

The conscientiousness trait reflects the tendency to follow rules, and it epitomizes ability, organization, self-discipline, and pursuit of achievement. Research has demonstrated that conscientiousness is positively correlated with restrained eating style (i.e., conscious control of food intake) and negatively correlated with an emotional eating style (Keller and Siegrist, 2015). Similarly, individuals with high levels of conscientiousness are more likely to wear seat belts, exercise consistently, sleep sufficiently, consume fruits and vegetables, and are less likely to indulge in cigarettes and alcohol (Raynor and Levine, 2009). It is acceptable to conclude from these studies that conscientiousness is highly correlated with engaging in health-promoting lifestyles. Besides, conscientious people are concerned about their health and have strong beliefs about self-management of health. Specifically, people high in conscientiousness have a high level of health self-efficacy. Further, Health-related self-efficacy accounts for more than 50% of the variance in health behavior (AbuSabha and Achterberg, 1997). We propose the following hypotheses:

H3: Conscientiousness is significantly and positively associated with the adoption behavior intention of healthcare information.

H4: Health concern and health-related self-efficacy mediate the relationship between conscientiousness and the adoption behavior intention of healthcare information. The relationship between independent variables and mediator variables is assumed as follows:

H4a: Conscientiousness is significantly and positively associated with health concerns.

H4b: Conscientiousness is significantly and positively associated with health-related self-efficacy.

Neuroticism is defined as emotional stability; individuals high in neuroticism typically exhibit anxiety, irritability, and depression (Digman, 1997). They are usually shy and tend to be easily embarrassed. people who exhibit high neuroticism are more likely to experience negative emotions such as worry and insecurities (Watson et al., 1994). Information that inappropriately describes disease risk would aggravate their negative emotions; that is, they perceive a high level of disease threat and tend to adopt healthcare information for risk avoidance. The higher the level of neuroticism, the greater the level of perceived disease threat, and thus the greater the tendency to adopt the healthcare information. Consequentially, we propose the following hypothesis:

H5: Neuroticism is significantly and positively associated with the adoption behavior of healthcare information.

H6: Perceive disease threat mediates the relationship between neuroticism and the adoption behavior intention of healthcare information. The relationship between independent variables and mediator variables is assumed as follows:

H6a: Neuroticism is significantly and positively associated with perceived disease threats.

Openness reflects an inclination toward intellectuality, which represents traits such as critical thinking, novelty, esthetics, and curiosity, and is related to imagination, morality, and creativity. Individuals with high levels of openness are open to new ideas and then utilize the evidence extracted to make judgments and decisions rather than relying too much on prior knowledge. They are less susceptible to anchoring biases as well (Haran et al., 2013). Alternatively, individuals with high openness are more likely to show an invitational attitude towards new, challenging ideas than those with moderate or low openness (Heinstrom, 2003b); hence, we propose the following hypothesis:

H7: Openness is significantly and positively associated with the adoption behavior intention of healthcare information.

### Moderating effect of social support theory

Social support is a concept mainly related to psychological health. It can provide individuals with positive emotional experiences in the form of regulating negative emotions, such as mitigating work stress in the social network and assisting individuals to attain emotional satisfaction, which is beneficial in terms of improving self-worth and imbuing a sense of psychological well-being. Overall, social support is divided into objective support and subjective spiritual support. Objective support indicates the material and other forms of tangible support, while subjective spiritual support refers to the individual’s perceived emotions regarding respect and love. The classic classifications of social support are generally informational support, emotional support, self-esteem support, substantive support, and network support (Braithwaite et al., 1999).

Additionally, studies have witnessed a strong relationship between psychological health and social support. People do not have access to substantive support in social networking environments. Similar to “face-to-face” social support, computer-mediated social support may also benefit those dealing with health issues and help in providing relief in stressful and depressing situations (Arora et al., 2007). Subjective spiritual support obtained online refers to self-esteem support and emotional support. Self-esteem support includes information that helps recipients reconstruct their self-concept and self-approval (Oh et al., 2013). Emotional support provides information that contains encouragement and empathy, making the recipients feel cared for and loved, and it is designed to help individuals reduce stress and negative influences. Positive emotionality is shown to enhance the likelihood of information acquisition (Heinström, 2006). In other words, perceived mental support may have a positive impact on information adoption. The following two hypotheses for this research are put forth:

H8: Perceived self-esteem support positively moderates the influence of personality traits on the adoption behavior intention of healthcare information.

H9: Perceived emotional support positively moderates the influence of personality traits on the adoption behavior intention of healthcare information.

**FIGURE 1:**
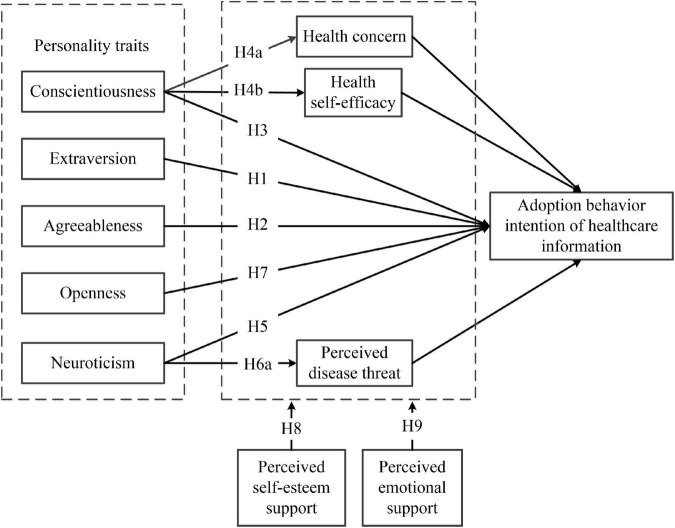
Theoretical model.

Based on the theoretical foundation of the present study, we considered the influence of thought, emotion, and behavioral characteristics for each personality trait on the adoption behavior intention of healthcare information and construct a theoretical model as illustrated in Figure 1.

## Research design and methods

### Research design

According to the research model in Figure 1, we drew on the existing literature and widely used scales to compile a measurement questionnaire. The confirmatory factor analytic approach is applied to test the hypotheses model. SPSS software was used for descriptive analysis and AMOS software was used for path analysis which is a statistical method for structural equation modeling. First of all, the SPSS process was used to test the moderated mediation effect, which is the validation of H4 and H6 and the partial test of H8 and H9. Next, we created interaction terms between perceived self-esteem support and perceived emotional support with personality traits. We then performed a regression analysis to test the interaction effects; that is, to further validate H8 and H9.

### Materials and methods

#### Subjects

The research mainly used questionnaires to obtain data. The researchers interacted with members of the online healthcare communities and distributed the questionnaires to them to obtain first-hand survey data. The specific survey process was as follows: six graduate students in the research group participated in posting, replying, commenting, and engaging in other forms of interactions on “Sweet Home,” an online diabetes community in China, “Baidu Insomnia Bar,” which belongs to the world’s largest Chinese community, “Baidu Post Bar,” health and diet topic section in “Zhihu,” a Chinese question and answer community, and sports and fitness group in “QQ,” a Chinese instant messaging tool. The data were obtained by directly asking questions from the active members of the community or by collecting paid questionnaires after researchers had gained a certain level of popularity. The duration of data acquisition process lasted for more than 5 months. Data were collected from 450 respondents out of 380 initial responses after excluding questionnaires that answers were found to be unreliable. Tables 1, 2 show the details of the questionnaire returns.

**TABLE 1:** Sample statistics for the questionnaire.

Data resources	Questionnaires issued	Returned questionnaires	Valid questionnaires	Effective rate
QQ groups	190	136	115	84.6%
Baidu post bars	150	131	110	84.0%
Zhihu	120	111	98	88.3%
Sweet home	100	72	57	79.2%
Total	560	450	380	84.4%

The values in the middle 3 columns are sample sizes.

**TABLE 2:** Sample statistics for the questionnaire.

Item	Category	Frequency of occurrence	Percentage
Gender	Boy	115	30.26%
	Girl	265	69.74%
Age	19 years old and below	27	7.10%
	20–29	46	12.11%
	30–39	47	12.37%
	40–49	73	19.21%
	50–59	83	21.84%
	60 years old and above	104	27.37%
Educational level	Middle school and below	73	19.21%
	High school	117	30.79%
	Junior college	68	17.89%
	Bachelor degree	92	24.21%
	Master degree and above	30	7.90%

The descriptive analysis of the sample showed that the female proportion was higher than male (2.3:1). The age of the sample was mainly concentrated in over 50 years old, accounting for 49.21% of the total, with relatively few adolescents and young adults. The elderly are the main users of the online health community because if the young people are uncomfortable, they will go directly to the hospital to register for medical treatment. The elderly over 60 years old are more concerned about their physical health, hoping to understand their physical health from multiple channels, and they also have more time to browse relevant information online, which is more in line with the actual situation. The educational level of the sample covered junior high school and below, senior high school, college, bachelor’s degree, and master’s degree and above. In general, the participants were older, and their level of education was not very high. Most of the participants had not received medical professional knowledge or training, and hence the sample was closely related to the real situation.

#### Measures

The questionnaire used in the present research was designed based on the research model and measurement scales. The questionnaire included measures of demographic and measures assessing 11 model variables shown in Figure 1 –five personality dimensions, health concern, health-related self-efficacy, perceived disease threats, perceived emotional support, perceived self-esteem support, and adoption behavior intention of healthcare information.

The emic-etic issue in personality assessment is often discussed. The etic approach assumed that trait measures adapted from one culture were adequate and sufficient representatives of the personality dimensions in another culture (Cheung et al., 2001). According to Yik and Bond (1993), emic and etic factors might “cut the social-perceptual world differently.” Nevertheless, there are studies that supported the generalizability of the Big Five trait taxonomy. The common measure of the Big Five factors is the NEO-Personality-Inventory, which has been applied cross-culturally (Cheung et al., 2001). Furthermore, the study of McCrae et al. (1996) confirmed the generalizability of the Big Five trait taxonomy to Chinese culture. Another study analyzed the internal consistency and factor structure of the Chinese Revised-NEO-Personality-Inventory, and the results suggested that five-factor model was identifiable in the Chinese sample (Leung et al., 1997). The personality trait scale of this research refers to the simplified version of the Big Five personality questionnaire compiled by Wang et al. (2010), which conforms to the language habits of Chinese and has good reliability and validity. The questionnaire divides personality into 5 dimensions with a total of 40 items using a six-point scale ranging from “very inconsistent” to “very consistent.” The reliability coefficients for each dimension of the short-form scale are good, with an average internal consistency coefficient of 0.836. Considering that the quality of the questionnaire would be degraded if there were too many items, researchers conducted a pre-survey of 160 participants and then used Amos to calculate the standardized regression weight of indicators for all personality dimensions. The items with high factor loadings were selected and 20 items were reserved. The remaining items showed high internal consistency, as the average Cronbach’s alpha score was 0.847. For the full scale, Table 3 shows the factor loadings for each model variable, reliability scores, and validity scores. Cronbach’s alpha and the CR (Composite Reliability) were used to estimate reliability. The recommended value for both of them is 0.8 or higher (Nunnally, 1978), with 0.6 or higher being within the acceptable range (Fornell and Larcker, 1981). The AVE (average variance extraction) is used for the evaluation of convergent validity. If the AVE is above 0.5, it represents that more than 50% of the variance of the observed variables is described, which means variables have good convergent validity. In particular, 0.36–0.5 is a barely acceptable standard for AVE in consideration of the actual orientation of data (Fornell and Larcker, 1981). Discriminant validity is demonstrated if the square value of AVE is greater than the correlation coefficient.

**TABLE 3:** AVE and CR values for model variables.

Variables	Encodings	Standardized factor loading	AVE	CR	Cronbach’s α
Health concern	HC1	0.742	0.537	0.826	0.866
	HC2	0.738			
	HC3	0.548			
Health self-efficacy	HSE1	0.687	0.518	0.834	0.838
	HSE2	0.752			
Perceived disease threat	PDT1	0.722	0.578	0.846	0.845
	PDT2	0.826			
	PDT3	0.751			
	PDT4	0.739			
Perceived esteem support	PES1	0.793	0.509	0.825	0.802
	PES2	0.682			
	PES3	0.657			
Adoption behavior intention	AB1	0.711	0.576	0.844	0.861
	AB2	0.771			
	AB3	0.746			
	AB4	0.805			
Neuroticism personality	NP1	0.782	0.669	0.890	0.888
	NP2	0.825			
	NP3	0.857			
	NP4	0.807			
Conscientiousness personality	CP1	0.683	0.576	0.853	0.860
	CP2	0.685			
	CP3	0.659			
	CP4	0.692			
Agreeableness personality	AP1	0.605	0.608	0.868	0.857
	AP2	0.716			
	AP3	0.620			
	AP4	0.680			
Openness personality	OP1	0.723	0.567	0.824	0.868
	OP2	0.652			
	OP3	0.714			
	OP4	0.737			
Extraversion personality	EP1	0.741	0.619	0.866	0.896
	EP2	0.855			
	EP3	0.805			
	EP4	0.741			

Health concern was measured using three items derived from a previous study by Oh et al. (2013). It was measured using a five-point Likert scale ranging from “disagree very much” to “agree very much.” The items demonstrated acceptable validity as well as good internal consistency, as Cronbach’s alpha score was 0.866.

A four-item scale taken from Becker et al. (1974) and Rosenstock (1974) was used to measure perceived disease threats. Three items in terms of perceived disease threats were selected from the study of Rosenstock (1974) after removing one item which is highly correlated with other items in an effort to cut down the length of the scale. Another item “I feel that my unhealthy lifestyle is threatening my health and that of my family,” was closely followed by the definition of perceived susceptibility in study of the Becker et al. (1974). Responses were provided using a five-point Likert ranging from “disagree very much” to “agree very much.” The items demonstrated good validity as well as high internal consistency, as the Cronbach’s alpha score was 0.845.

To measure health-related self-efficacy, a two-item was developed closely following the later definition for self-efficacy proposed by Lee et al. (2008), measuring the “individuals’ beliefs about their ability to manage their health.” The items used five-point Likert ranging from “disagree very much” to “agree very much.” The Cronbach’s alpha score was 0.838. AVE value was greater than the recommended value. Therefore, the items demonstrated acceptable reliability as well as validity.

Perceived emotional support was measured with three items derived from the study by Johnson and Lowe (2015), using a five-point Likert ranging from “disagree very much” to “agree very much.” Seeing that Cronbach’s alpha score was 0.820, the items exhibited good internal consistency.

Perceived self-esteem support was measured with three items taken from the research done by Rosenberg (1965). This measure did not cover all items Rosenberg originally suggested. Items that are concise and easy to understand were reserved and adapted by phrasing them in forms of health-related self-esteem. Items were measured using a five-point Likert scale ranging from “disagree very much” to “agree very much.” The items presented good validity as well as internal consistency, as Cronbach’s alpha score was greater than 0.802.

To measure the adoption behavior intention, a four-item were taken from Cheung et al. (2008), and the items were adapted in forms of health-related issues. Items were measured using a 5-Likert scale ranging from “disagree very much” to “agree very much.” The Cronbach’s alpha score was 0.861, which suggested good internal consistency. Convergent validity was also verified as the AVE of measurement items was all within an acceptable range. Table 4 shows the correlation coefficients between the factors and their corresponding AVE square roots. The correlation coefficients of the factors, the bottom half of the matrix, were less than the square root of the AVE, i.e., diagonal elements were larger than off-diagonal elements, which indicated that the questionnaire had strong discriminant validity. In summary, the whole scale had good reliability and validity.

**TABLE 4:** The correlation coefficients between the variables and their corresponding AVE square roots.

Variables	1	2	3	4	5	6	7	8	9	10	11
HC	0.733										
HSE	0.486***	0.720									
PDT	0.085**	−0.347**	0.760								
PES	0.357**	0.354***	−0.098**	0.796							
PSS	−0.156**	−0.474**	0.442**	−0.161**	0.713						
AB	0.505***	0.359***	0.034**	0.600***	−0.084**	0.759					
NP	−0.063**	−0.433***	0.565***	−0.078**	−0.703***	0.067**	0.818				
CP	0.312***	0.719**	−0.114**	0.255**	0.425***	0.033***	−0.411***	0.759			
AP	0.157***	0.270**	−0.067**	00.211**	−0.113**	0.024***	−0.202**	0.388**	0.780		
OP	0.920**	0.404***	−0.067**	0.201**	−0.131**	0.245**	−0.137***	0.457***	0.437***	0.753	
EP	0.274**	0.569***	−0.194**	0.272***	−0.525***	0.252**	−0.49***	0.446***	0.409***	0.599***	0.787

The diagonal line is the square root of the AVE value of each variable (***p < 0.001, **p < 0.01; HC, Health Concern; HSE, Health-related Self-Efficacy; PDT, Perceived Disease Threats; PES, Perceived Emotional Support; PSS, Perceived Self-esteem Support; AB, Adoption behavior intention; NP, Neuroticism; CP, Conscientiousness; AP, Agreeableness; OP, Openness; EP, Extraversion).

## Data analysis

### Test for the direct effect of personality traits on the adoption behavior intention of healthcare information

We used Amos (version 22.0) to create 9 latent variables, including five personality dimensions, health concern, health-related efficacy, perceived disease threats, and adoption behavior intention of healthcare information, then used Amos to draw the corresponding structural equation model. In order to test the structural validity of the model, confirmatory factor analysis was conducted (Rafiq et al., 2022). All the fit indices shown in Table 5 are used to test the fit of the model to the actual data, and they are all within the recommended range. Therefore, the actual data fit the model well in general.

**TABLE 5:** The degree of model fit.

Indices of model fit	Result	Criterion of fitness
χ2/df	1.674	<3
GFI	0.916	>0.9
RMSEA	0.046	<0.05
IFI	0.923	>0.9
CFI	0.922	>0.9
TLI	0.913	>0.9

Table 6 presents the results of the path analysis. It can be concluded that health concerns and health-related self-efficacy have significant effects on the adoption behavior intention of healthcare information. H1, H2, H3, H5, and H7 are supported by the significant and positive impact of extraversion, agreeableness, conscientiousness, neuroticism, and openness on the adoption behavior intention of healthcare information.

**TABLE 6:** Standardized path coefficients and results of the hypothesis test.

Path	Estimate	S.E.	*p*
Adoption behavior ← Health concerns	0.406	0.068	***
Adoption behavior ← Health-related self-efficacy	0.494	0.192	***
Adoption behavior ← Perceived disease threat	0.027	0.056	***
Adoption behavior ← Neuroticism	0.217	0.052	***
Adoption behavior ← Conscientiousness	0.106	0.184	***
Adoption behavior ← Agreeableness	0.022	0.058	***
Adoption behavior ← Openness	0.114	0.056	*
Adoption behavior ← Extraversion	0.132	0.049	**
Health concern ← Conscientiousness	0.520	0.086	***
Health self-efficacy ← Conscientiousness	0.737	0.087	***
Perceived disease threat ← Neuroticism	0.481	0.055	***

***p < 0.001, **p < 0.01, *p < 0.05.

### Test for mediating and moderating effects

#### Test for the moderated mediation model

In the selection of mediating effect test methods, most scholars mainly follow the test method of causal stepwise regression proposed by Baron et al. But in recent years, many scholars have questioned the B-K method (Chen et al., 2013). Subsequently, Baron et al. also recommended the Sobel test which can directly test the mediating effect. However, the validity of the Sobel test still has major drawbacks (Mackinnon et al., 2004). This manuscript uses the Bootstrapping test method proposed by Taylor et al. (2008) to test the mediating effect of health concern and health-related self-efficacy in the path “conscientiousness → adoption behavior intention of healthcare information” and the mediating effect of perceived disease threat in the path “neuroticism → adoption behavior intention of healthcare information.”

While using “model4” (i.e., a simple mediation model) in the SPSS macro compiled by Hayes and controlling for age and gender variables, using a repeated random sampling method, 2000 Bootstrap samples were selected from the original data (*n* = 380), and the bias-corrected Boot-Strap procedure was used to test the mediating effects of health concern and health-related self-efficacy in the relationship between conscientiousness and adoption behavior intention of healthcare information.

The results verified that conscientiousness was a significant predictor of health concerns and health-related self-efficacy. Thus, H4a and H4b were supported. Table 7 outlines the total and direct effect values of the impact of conscientiousness on the adoption behavior intention, as well as the mediating effect values for both health concern and health-related self-efficacy in the relationship between conscientiousness and adoption behavior intention. The result affirmed the mediating effect, which supported H4. Besides, the direct effect of conscientiousness on adoption behavior intention was significant.

**TABLE 7:** Decomposition of the total, direct, and mediating effects.

	Effect value	Standard error	BootLLCI	BootULCI	Relative effect value
Total effects	0.335	0.056	0.227	0.449	
Direct effects	0.177	0.052	0.084	0.121	52.84%
Mediating effect of health concern	0.134	0.031	0.081	0.206	40.00%
Mediating effect of health-related self-efficacy	0.183	0.041	0.102	0.264	54.63%

Effect value refers to the values estimated by bootstrap; The standard error refers to the standard error of the indirect effect estimated by the bootstrap method, BootLLCI, and BootULCI are the upper and lower bounds of the 95% confidence interval; Relative effect value refers to the percentage of direct or indirect effects on the total effect.

**FIGURE 2:**
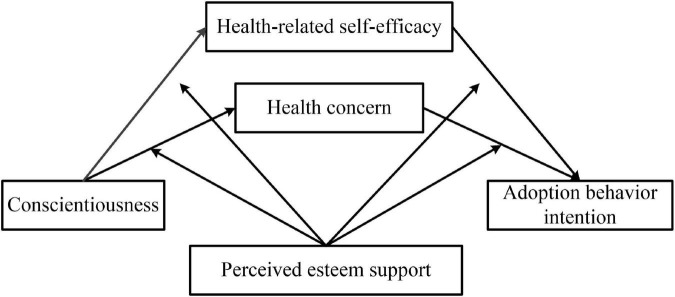
The moderated mediation model.

Second, relying on “model58” (model58 assumes that the first half and the second half of the mediation model are both affected by the same moderating variable) in the SPSS macro compiled by Hayes while controlling for age and gender variables allowed us to elicit the bootstrap approach to test how the relationship between conscientiousness and adoption behavior intention was mediated by health concerns and health-related self-efficacy. Also, how it was moderated by social support. Table 8 specifies that the impact of perceived self-esteem support on the relationship between conscientiousness and adoption behavior intention was significant overall (the *p*-value of 4 interaction items were all less than 0.05). Specifically, perceived self-esteem support significantly and positively moderated the link between conscientiousness and health concern, conscientiousness and health-related self-efficacy, and health-related self-efficacy and adoption behavior intention, and it negatively moderated the association between health concern and adoption behavior intention. Briefly, the moderated mediation model illustrated in Figure 2 was confirmed. Additionally, the result of regression analysis, viewable in Table 9, indicated that perceived emotional support did not have a significant impact on the relationship between conscientiousness and adoption behavior intention of healthcare information; that is, mediating effect was only moderated by perceived self-esteem support.

**TABLE 8:** Test for the mediating effect of health concern and health-related self-efficacy moderated by perceived self-esteem support.

	Health concern	Health self-efficacy	Adoption behavior
Gender	0.012	0.025	–0.001
Age	0.049	0.043	−0.087*
Edu	−0.083*	–0.043	0.001
Med	–0.160	–0.208	–0.077
CP	0.328***	0.539***	0.127*
PSS	–0.085	0.005	−0.208***
HC			0.437***
HSE			0.321***
Product of CP and PSS	0.190**	0.121*	
Product of HC and PSS			−0.274***
Product of HSE and PSS			0.180*
*R*-squared	0.153	0.573	0.749
*F*	6.584	17.702	32.092

Values in middle 3 columns refers to the regression coefficient; ***p < 0.001, **p < 0.01, *p < 0.05.

**TABLE 9:** Test for the mediating effect of health concern and health-related self-efficacy moderated by perceived emotional support.

	Health concern	Health self-efficacy	Adoption behavior
Product of CP and PES	0.034	–0.013	
Product of HC and PES			–0.05
Product of HSE and PES			–0.047
*R*-squared	0.244	0.573	0.749
*F*	11.697	17.702	32.092

Values in middle 3 columns refer to the regression coefficient.

Moreover, the association between conscientiousness and adoption behavior intention mediated by health concerns tended to be weaker as the level of perceived self-esteem support was higher. Likewise, the association between conscientiousness and adoption behavior intention mediated by health-related self-efficacy tended to be stronger as the level of perceived self-esteem support was higher.

Finally, the same method was adopted to test the mediating effect of perceived disease threats in the relationship between neuroticism and the adoption behavior intention. The results showed that neuroticism was a significant predictor of perceived disease threats, which indicated that H6a was supported. At the same time, perceived disease threat plays a significant mediating role, which supports H6.

#### Test for moderating effects

There are three methods to test the moderating effect, according to the different methods of independent variables and moderating variables, the independent variables and moderating variables in this manuscript are classified variables, which is actually the interaction significance analysis in multivariate analysis of variance, so we can use the linear regression method in SPSS24.0 to test moderating effects, we centered the variables of personality traits, perceived self-esteem support and perceived emotional support, and then created interaction terms to form new variables. Regression analysis was then conducted between the interaction and dependent variables to test the moderating effect of perceived emotional support and perceived self-esteem support on the relationship between four remaining personality dimensions and the adoption behavior intention of healthcare information–extraversion, agreeableness, neuroticism, and openness, which was a partial test of H8 and H9.

As demonstrated in Table 10, perceived self-esteem support significantly and negatively moderated the relationship between neuroticism and the adoption behavior intention. The results also found that the impact of neuroticism on adoption behavior intention was significant only at a low level of perceived self-esteem support. In addition, perceived self-esteem support significantly enhanced the direct impact of conscientiousness on adoption behavior intention. However, the impact of perceived self-esteem support on the relationship between the remaining three personality traits, extraversion, agreeableness, openness, and the adoption behavior intention was not verified.

**TABLE 10:** Test results of moderating effects of perceived self-esteem support.

Interaction items	Standardized regressive coefficient β
Product of extraversion and perceived self-esteem support	–0.020
Product of agreeableness and perceived self-esteem support	0.019
Product of conscientiousness and perceived self-esteem support	0.144*
Product of neuroticism and perceived self-esteem support	−0.255***
Product of openness and perceived self-esteem support	0.033

***p < 0.001, *p < 0.05.

**FIGURE 3:**
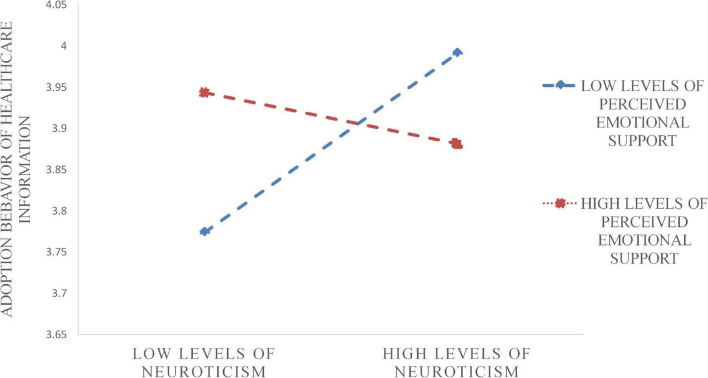
Moderating effect of PES in the relationship between NP and AB.

**FIGURE 4:**
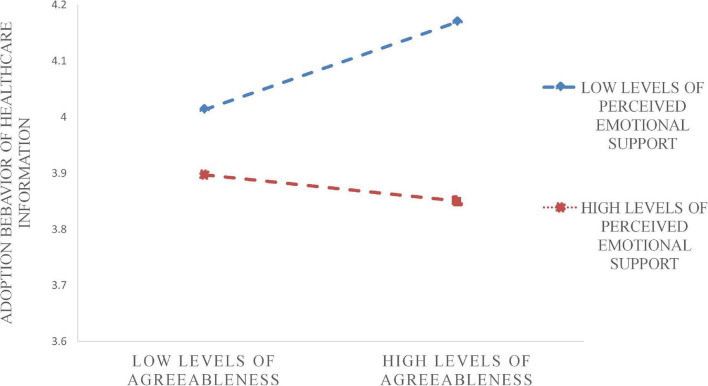
Moderating effect of PES in the relationship between AP and AB.

Table 11 shows the significant level of moderating effect of perceived emotional support. It also can be observed visually from Figures 3, 4 that perceived emotional support weakened the relationship between two personality traits, agreeableness and neuroticism, and adoption behavior intention.

**TABLE 11:** Test results of moderating effects of perceived emotional support.

Interaction items	Standardized regression coefficient
Product of extraversion and perceived emotional support	–0.049
Product of agreeableness and perceived emotional support	−0.152*
Product of conscientiousness and perceived emotional support	–0.040
Product of neuroticism and perceived emotional support	−0.131**
Product of openness and perceived emotional support	–0.070

**p < 0.01, *p < 0.05.

In summary, perceived self-esteem support positively moderated the relationship between conscientiousness and the adoption behavior intention, and negatively moderated the relation between neuroticism and adoption behavior intention. H8 was partially supported. Perceived emotional support weakened the relationship between two traits, agreeableness and neuroticism, and the adoption behavior intention. H9 was not supported. In addition, all hypotheses and the results of the study are exhibited in Table 12.

**TABLE 12:** Hypotheses and results.

Hypotheses	Effects to be analyzed	Results
1	Effect of extraversion on adoption behavior intention	Supported
2	Effect of agreeableness on adoption behavior intention	Supported
3	Effect of conscientiousness on adoption behavior intention	Supported
4	Mediation effect of health concern and health-related self-efficacy between conscientiousness and adoption behavior intention	Supported
5	Effect of neuroticism on adoption behavior intention	Supported
6	Mediation effect of perceived disease threats between neuroticism and adoption behavior intention	Supported
7	Effect of openness on adoption behavior intention	Supported
8	Moderation effect of perceived self-esteem support on the relation between personality traits and adoption behavior intention	Partially supported
9	Moderation effect of perceived emotional support on the relation between personality traits and adoption behavior intention	Unsupported

## Discussion

### The impact of personality traits on the adoption behavior intention of healthcare information

The present study reveals the positive effect of extraversion, agreeableness, conscientiousness, neuroticism, and openness on the adoption behavior intention and indicates the positive significance of health concern and health-related self-efficacy in mediating the association between conscientiousness and adoption behavior intention. The result of this part was consistent with the assumptions set forth above. Positive emotionality may be a possible explanation for the result regarding extraversion. Previous research has demonstrated that positive emotionality and high motivation enhance receptivity to information acquisition (Heinström, 2006). On the other hand, positive emotionality may make individuals with high levels of extraversion interact with others more frequently. Such frequent interaction may facilitate the adoption behavior intention. This finding suggests that creating the conditions to produce positive and highly motivating emotions may contribute to information handling.

People with agreeableness personalities are more receptive to information published on different platforms and more willing to share knowledge on platforms (Yang and Li, 2021). They also tend to be more receptive to health messages posted by online health communities.

The direct impact of conscientiousness on adoption behavior intention was also verified. It is assumed that people are taking more responsibility for their own bodies and are willing to take community advice to help them achieve better health. Besides, the indirect effect of this trait on adoption behavior intention was verified through two mediator variables–health concern and health-related self-efficacy, which is the one of main findings of the study. The finding further suggests that paying more attention to health-related information and increasing the sense of health-related self-efficacy can be conducive to the understanding and acquisition of information, which further benefits the performance of health management. The result is partially consistent with the finding of the previous study (Lu et al., 2014) suggesting that conscientiousness and agreeableness have an indirect impact on the adoption of information technologies in healthcare management.

Lee and Kurtz (2019) found that neurotic individuals were more inclined to experience negative affect after viewing health promotion messages. Negative emotions reduce the ability to make judgments and decisions as negative emotions consume energy and distract concentration (Heinstrom, 2003b). Therefore, people high in neuroticism have a subconscious tendency to follow the viewpoint. Likewise, Lee and Kurtz (2019) assumed that the connection between negative affect and neuroticism may lead to impulsivity or the tendency to act rashly. Negative emotions should be consciously controlled to avoid being induced by extreme arguments, such as maladaptive information regarding diet control. Moreover, the present study bolsters the finding of Lee and Kurtz (2019) by confirming the positive associations between neuroticism and perceived disease threats, which partially explained the origin of negative affect. However, the role of perceived disease threats in mediating the relationship between neuroticism and adoption behavior intention was not significant, so the mechanisms through which neuroticism influences adoption behavior intention need to be further explored.

As for openness, Individuals who score higher on the trait of openness may not arbitrarily take a confrontational approach towards emerging ideas that do not confirm old knowledge and tend to be early adopters of the information due to the fact that they like to explore more possibilities. Being invitational to novel ideas and embracing a new healthy lifestyle may help to promote physical and mental health.

Overall, all the personality traits positively influenced the willingness to adopt healthcare information. Contact the specific life, product managers in the healthcare realm can establish a customer-oriented community and classify them according to personality traits. Supportive messages, such as compliments on efforts to improve health and strong identification with other users, can be disseminated to facilitate the information adoption of customers who score higher in traits of conscientiousness and neuroticism.

### Moderating effect of social support

#### Moderating effect of perceived self-esteem support

The present study found that a low level of perceived self-esteem support strengthened the relationship between neuroticism and adoption behavior intention of healthcare information. Neuroticism is negatively associated with self-esteem (Watson et al., 2002), and self-esteem is negatively correlated with avoidance goals (Heimpel et al., 2006). Additionally, people who are more disposed towards the avoidance orientation are also more likely to be persuaded by high-threat information (van’t Riet et al., 2012). Therefore, individuals with high neuroticism are likely to be persuaded by threatening information that harms their sense of self-worth.

The study also found that perceived self-esteem support significantly and positively moderated the relationship between conscientiousness and adoption behavior intention of healthcare information. Further, perceived self-esteem support had a significant and positive impact on three paths of the dual mediation model present in Figure 2: the path of conscientiousness to a health concern, the path of conscientiousness to health-related self-efficacy, and the path of health-related self-efficacy to adoption behavior intention. The improvement of self-worth and the strengthening of self-identity may enhance their belief in personal health management. The willingness to adopt information is also enhanced in response. This result is consistent with the findings of Oh et al. (2013) and Deng and Liu (2017) stating that self-esteem support positively affects an individual’s health-related self-efficacy.

In addition, perceived self-esteem support weakened the relationship between health concerns and adoption behavior intention. The possible explanation maybe the partial shift in the motivation for information acquisition from health concerns to perceived benefits of the information (i.e., the emotional value from increased self-identity and sense of worth as well as the value of the information itself). Consequently, the study confirmed the positive impact of perceived self-esteem support concerning the adoption behavior intention of healthcare information. Self-esteem depends on social influence (Baumeister, 2013), which means this type of social support is common in communities. People who are not satisfied with their physical condition may feel stuck with pessimistic emotions and they can seek social support from an online healthcare community.

From the perspective of individuals, it is encouraged to become a healthcare information literate life-long learner. Developing positive traits can be learned to a certain extent through the attitude of invitational and openness to information and prudence in considering advice. In addition, interaction and consultation along with positive emotions may enhance the understanding of information and accelerate the process of problem-solving, while negative emotions may be an obstacle to successful database searches (Heinstrom, 2003a). In particular, an increase in self-esteem is highly desirable, which can help avoid being induced purposefully and improve self-efficacy as well.

#### Moderating effect of perceived emotional support

The results revealed that perceived emotional support negatively moderated the relationship between neuroticism and agreeableness and the adoption behavior intention of healthcare information. Agreeableness is associated with caring: people who are high in agreeableness are more likely to pay attention to negative emotional information while caring for and showing friendliness to others. As a consequence, they are less likely to give adequate attention to the required information. Similarly, the vulnerability to negative emotions of neuroticism could explain the result that perceived emotional support weakened its impact on adoption behavior intention. In general, esteem supportive information can indeed benefit users in the community.

The results did not indicate a significant effect of perceived emotional support on the relationship between extraversion, conscientiousness, and openness and the adoption behavior intention of healthcare information. These personality traits significantly predict information skills (Song and Kwon, 2012). Therefore, people high in these traits may pay more attention to the quality and utility of the information than emotional information. In other words, emotional information may be irrelevant to those who possess excellent information competency.

## Conclusion

Based on the Big Five personality and social support theory, this study examined the influence of Big Five personality traits on adoption behavior intention on healthcare information in online communities as well as the effect of social support on the relationship between them. The findings of this study can help to grasp the basic rules of user adoption behavior intention in healthcare communities and have important practical implications for guiding and optimizing user information adoption behavior. Specifically, users can consciously screen out content that tempts them to adopt information described in inappropriate words. In addition, this study enriches the research in the field of information adoption by steering the study from the perspective of informational cognitive to individual cognitive on factors affecting the adoption behavior intention of healthcare information. The study also has practical value. For instance, this finding is conducive to the optimization of user insight. Gaining insight into users purely based on a few simple attributes is ill-considered; the addition of personality traits can be a feasible optimization scheme.

## Limitations and further research

This study has some limitations in terms of research content and methodology. First, the effects of demographic variables on the relationship between personality traits and adoption behavior of healthcare information were not studied thoroughly. Second, as the geographic area covered by the participants in the data survey for this study was not adequate, the replicability of this study’s findings across different nationalities and cultural contexts needs to be further investigated. Finally, there was a lack of specific descriptive materials to comprehensively and deeply understand the influence mechanism of personality traits on adoption behavior intention. Finally, in-depth interviews with incentives can be used in future research to further analyze the role of personality traits in the behavioral intention to adopt healthcare information.

## Data availability statement

The original contributions presented in this study are included in the article/Supplementary material, further inquiries can be directed to the corresponding author.

## Ethics statement

Ethical review and approval was not required for the study on human participants in accordance with the local legislation and institutional requirements. Written informed consent for participation was not required for this study in accordance with the national legislation and the institutional requirements.

## Author contributions

YZ constructed the theoretical framework of the manuscript. HJ collected the data and made a statistical analysis with the help of ZZ. All authors contributed to the article and approved the submitted version.
