# Supplementary material for: Information adoption behavior in online healthcare communities from the perspective of personality traits
Source: Front Psychol. 2022 Oct 18;13:973522. doi: 10.3389/fpsyg.2022.973522 (PMC9623248; doi:10.3389/fpsyg.2022.973522)
Supplement: Supplementary file 1 [file Data_Sheet_1.DOCX]

Hi! We are a group of students from Nanchang University, and we are conducting a survey which constitutes a major part of our research project. Subsequently, we are going to ask you a few questions based on the purpose of understanding your attitude towards healthcare information in online communities. We sincerely hope that you can spend a few minutes on this survey, and we would definitely value your opinions! Your identity and answers are absolutely confidential!

Note :1. **Healthcare information** refers to the information related to health promotion means, including disease prevention, mental health, dietary nutrition, lifestyle, sleep, weight loss, fitness and muscle gain, skin care, sexual health, traditional Chinese medicine and other daily healthcare knowledge.

2. **Online community**: your current forum/group/Q&A platform/post bar.

Q1: your gender is ()

1.Boy

2.Girl

Q2: WHAT is your age? ()

1. 19 years old and below
2. 20 to 29
3. 30 to 39
4. 40 to 49
5. 50 to 59
6. 60 years old and above

Q3: What is your level of education? ()

1. middle school and below
2. high school
3. junior college
4. Bachelor Degree
5. Master degree and above

Q4: Are you medically educated?

1.Yes

2. no

The remaining subjects are presented in terms of table, and their options are illustrated in the manuscript.

Table 1

| variables | code | items |
| --- | --- | --- |
| Health Concern  (HC) | HC1 | I often collect health information improve my own or my family's health. |
|  | HC2 | I often get health information through the online community. |
|  | HC3 | I actively seek the advice of others when I encounter health problems. |
| Health Self-Efficacy  (HSE) | HSE1 | Even when there are difficulties, I can overcome them and follow my doctor's advice to improve my health. |
|  | HSE2 | I am confident that I will be able to seek medical assistance related to my illness. |
| Perceived Disease Threat  (PDT) | PDT1 | I think I’m more likely to get sick than others. |
|  | PDT2 | I feel my health is deteriorating. |
|  | PDT3 | I think I will most likely suffer from chronic diseases in the future. |
|  | PDT4 | I feel that my unhealthy lifestyle is threatening my health and that of my family. |
| Perceived Esteem Support  (PES) | PES1 | I feel I do not have much to be proud of when I am in online healthcare community. |
|  | PES2 | I wish I could have more respect for myself after reading some healthcare information in the community. |
|  | PES3 | On the whole, I am satisfied with my appearance after reading some healthcare information in the community. |
| Adoption behavior  (AB) | AB1 | I agree with the opinions obtained from the online healthcare community. |
|  | AB2 | I will actually apply the healthcare information or advice I get from the online community to my life. |
|  | AB3 | I will share the healthcare information obtained from the online community with my friends. |
|  | AB4 | I will get healthcare information from the online community regularly in the future. |

Table 2 Scale of personality traits

| Personality traits | code | items |
| --- | --- | --- |
| conscientiousness | CP1 | Once I set my goal, I will keep working hard to achieve it. |
|  | CP2 | I work very hard. |
|  | CP3 | I always try my best to do everything. |
|  | CP4 | Doing things logically and methodically is my hallmark. |
| openness | OP1 | I'm an adventurous, unconventional person. |
|  | OP2 | I like adventure |
|  | OP3 | I have a strong curiosity about many things. |
|  | OP4 | I am eager to learn something new, even if it has nothing to do with my daily life. |
| extraversion | EP1 | I get bored with parties full of people. |
|  | EP2 | In lively parties, I often act proactively and have fun. |
|  | EP3 | Occasions where I'm present usually don't get cold. |
|  | EP4 | I enjoy attending social and recreational gatherings. |
| agreeableness | AP1 | I often feel that the pain of others has nothing to do with me. |
|  | AP2 | Although there are some cheaters in the society, I think most people are trustworthy. |
|  | AP3 | I often feel sad when people tell me about their misfortunes. |
|  | AP4 | Despite the dark side of human society(e.g., war, evil, fraud), I still believe that human nature is generally good. |
| neuroticism | NP1 | Sometimes I feel like I'm worthless. |
|  | NP2 | I often worry about unimportant things |
|  | NP3 | I often feel unsettled inside. |
|  | NP4 | I often worry that something bad is going to happen. |
